# Organosoluble Starch-Cellulose Binary Polymer Blend as a Quasi-Solid Electrolyte in a Dye-Sensitized Solar Cell

**DOI:** 10.3390/polym12030516

**Published:** 2020-02-27

**Authors:** Vidhya Selvanathan, Rosiyah Yahya, Mohd Hafidz Ruslan, Kamaruzzaman Sopian, Nowshad Amin, Majid Nour, Hatem Sindi, Muhyaddin Rawa, Md. Akhtaruzzaman

**Affiliations:** 1 Solar Energy Research Institute (SERI), Universiti Kebangsaan Malaysia (UKM), Bangi 43600, Malaysia; hafidzruslan@gmail.com (M.H.R.); ksopian@ukm.edu.my (K.S.); 2 Department of Chemistry, Faculty of Science, University of Malaya, Kuala Lumpur 50603, Malaysia; rosiyah@um.edu.my; 3 Institute of Sustainable Energy, Universiti Tenaga Nasional (@The National Energy University), Jalan IKRAM-UNITEN, Kajang 43000, Malaysia; nowshad@uniten.edu.my; 4 Department of Electrical and Computer Engineering, King Abdulaziz University, Jeddah 21589, Saudi Arabia; mnour@kau.edu.sa (M.N.); hfsindi@kau.edu.sa (H.S.); 5 Centre of Research Excellence in Renewable Energy and Power Systems, King Abdulaziz University, Jeddah 21589, Saudi Arabia; mrawa@kau.edu.sa; 6 Department of Electrical, Electronic and Systems Engineering, Faculty of Engineering and Built Environment, The National University of Malaysia, Bangi 43600, Malaysia

**Keywords:** phthaloyl starch, hydroxyethyl cellulose, rheology, quasi-solid electrolyte, dye-sensitized solar cell

## Abstract

This work is a pioneer attempt to fabricate quasi-solid dye-sensitized solar cell (QSDDSC) based on organosoluble starch derivative. Rheological characterizations of the PhSt-HEC blend based gels exhibited viscoelastic properties favorable for electrolyte fabrication. From amplitude sweep and tack test analyses, it was evident that the inclusion of LiI improved the rigidity and tack property of the gels. On the other hand, the opposite was true for TPAI based gels, which resulted in less rigid and tacky electrolytes. The crystallinity of the gels was found to decline with increasing amount of salt in both systems. The highest photoconversion efficiency of 3.94% was recorded upon addition of 12.5 wt % TPAI and this value is one of the highest DSSC performance recorded for starch based electrolytes. From electrochemical impedance spectroscopy (EIS), it is deduced that the steric hindrance imposed by bulky cations aids in hindering recombination between photoanode and electrolyte.

## 1. Introduction

Dye-sensitized solar cell (DSSC) is a third generation photovoltaic device, developed first by O’Regan and Gratzel in 1991. The conceptualization of DSSC draws inspiration from photosynthesis in which chlorophyll only plays a role in light harvesting but does not participate in charge transfer [1]. Similarly, in DSSC, charge generation takes place at a semiconductor-dye interface while charge transport is performed by the semiconductor and electrolyte. As it is the case in any electrochemical device, electrolytes are one of the essential components in DSSC. Being mainly responsible for the internal charge transport between the electrodes in order to continually replenish the dye, electrolytes can directly influence the photovoltaic parameters.

Despite remarkable efficiencies attained upon using liquid electrolytes, the application of these electrolytes in commercialized DSSC was deterred by practical flaws such as mechanical instability, leakage, volatilization of solvent, and photodegradation of the dye [2]. On the other hand, the credentials of a completely solid electrolyte do not live up to the required performance often due to poor contact between electrodes [3]. The trade-off between electrochemical performance and mechanical stability of the electrolyte was achieved by introducing a new class of electrolytes, namely quasi-solid electrolyte (QSE). This unique state of the material is often achieved by the incorporation of allows the electrolyte to possess both the cohesive property of solid and diffusive property of liquid [4].

By far in literature, a vast array of synthetic polymers have been employed as the host in quasi-solid DSSC (QSDSSC) [5]. Parallel to this, novel ventures of utilizing natural polymers as alternative host material is emerging in recent publications. Starch is one of such natural polymers that hold a special potential as a gelling agent in QSE due to its inherent viscosifying properties. Application of starch as a polymer electrolyte in DSSC began with a simple fabrication technique in which starch was incorporated with inorganic salts or ionic liquids to elevate its conductivity [6,7]. Despite the presence of additives, the energy conversion efficiency of these systems did not exceed 1%. Thus in recent studies, a novel approach of chemically modifying starch via grafting or cross-linking reactions has been adapted prior to electrolyte fabrication [8,9]. However, these methods could not resolve the high crystallinity and low mechanical strength of starch based quasi-solid electrolytes.

Herein we propose a dual strategy to effectively convert starch into high efficiency QSDSSC. Firstly, starch is esterified into phthaloyl starch to synthesize an organosoluble derivative. This procedure aids in diminishing crystallinity of the polymer and hence allow easy transition of ions within its matrix [10]. Subsequently, phthaloyl starch is blended with hydroxyethyl cellulose, incorporating the adhesive nature of starch and structural stability of cellulose to form a tacky yet sturdy gel. Previously, we have analyzed the effect of different ratios of the starch and cellulose components in the blend and an optimum composition for DSSC application had been identified [11]. In this work, the performance of the fabricated QSDSSC is further optimized by preparing two series of gels comprising different quantities of lithium iodide and tetrapropylammonium iodide as the ionic species. The choice of cation used in the electrolyte composition has been found to significantly impact DSSC performance. Hence, various studies in literature have been dedicated to elucidate the role of the cations in factor such as the rate of the electron injection, electron lifetime in the photoanode, recombination rate, and electron diffusion coefficient [12,13,14]. Conversely, in this work, apart from electrochemical properties, the influence of the cationic species on the rheological properties (such as rigidity and tackiness) of the gels have also been analyzed in depth. This approach ensures the improvement in photovoltaic performances is not performed at the expense of the mechanical stability of the electrolyte. 

## 2. Materials and Methods 

### 2.1. Materials

Potato starch (M_w_ = 1,600,000) and tetrapropylammonium iodide (TPAI) were purchased from Omya Hamburg and Fisher Scientific, respectively. Phthalic anhydride, pyridine, hydroxyethyl cellulose (M_w_ = 720,000), lithium iodide, and dimethylformamide (DMF) were obtained from Merck. All materials were used as received.

### 2.2. Experimentals

#### 2.2.1. Phthaloylation of Starch

Phthaloyl starch was synthesized following the method reported previously [10]. Starch was suspended into a flask containing DMF and heated up to 80 °C under constant stirring. After 30 min, phthalic anhydride and pyridine were then added to the mixture, and the reaction was allowed to proceed for another 4 h. The phthaloylated starch product that was in a homogenous solution was then retrieved by precipitation with isopropanol and dried until constant weight. 

#### 2.2.2. Preparation of Electrolytes

**Figure 1:**
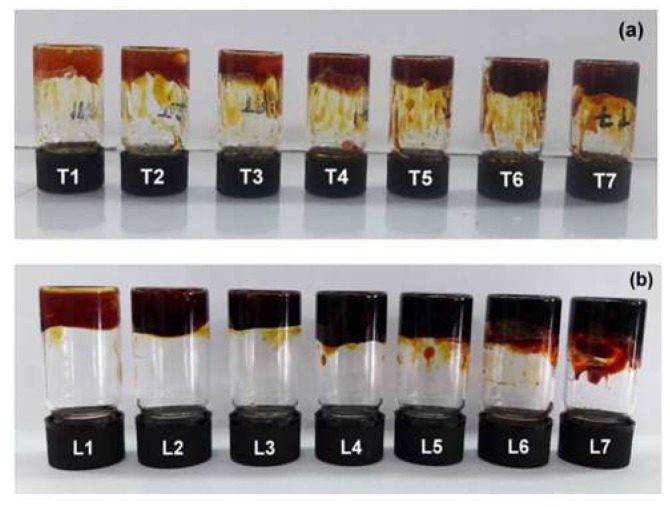
Photographs of PhSt-HEC-DMF gel electrolytes with (**a**) TPAI and (**b**) LiI.

The gel electrolytes were prepared by dissolving an appropriate amount of salt in DMF followed by addition of phthaloyl starch (0.28 g) and hydroxyethyl cellulose (0.12 g). This mixture was stirred at 70 °C for 6 h to form the quasi-solid gels. In all samples, the polymer to solvent weight ratio was fixed at 1:3. The detailed composition of the gels fabricated is listed in Table 1. Figure 1 depicts the photographs of PhSt-HEC-DMF gel electrolytes with TPAI and LiI respectively. 

### 2.3. Characterizations of Electrolytes

All rheological tests were performed using an Anton Paar Physica MCR 301 rheometer with parallel-plate geometry (2.5 cm diameter). Amplitude sweep tests of the gel samples were studied by recording the variations in storage modulus (G′) and loss modulus (G″) as a function of strain ranging from 0.1% to 250% at a measuring gap of 0.1 mm and an angular frequency of 10 rads^−1^. In order to gauge the adhesive property of the gels, a tack test analysis was also performed. For the tack test study, the experiments were done at three intervals. First, the fresh sample was placed on the bottom plate and the top plate was lowered to a measuring gap of 0.25 mm. The sample was then subjected to short shearing for 1 s with a shear speed of 1000 s^−1^. Finally, the top plate was removed vertically at a constant velocity of 5 ms^−1^ and the normal force experienced by the top plate was recorded. As the top plate was removed, the sample pulls on the measuring system and therefore the measured normal force values were negative. Operation of the rheometer and analysis of the rheological parameters were carried out using the Rheoplus/32 V3.60 software.

To confirm the effect of the polymer blending on the crystallinity of gels, X-ray diffraction (XRD) spectra of the samples were recorded on a PANalytical X’Pert^3^ MRD diffractometer. The gel samples were uniformly applied on the surface of the sample holder, which was then placed in the diffractometer and the samples were directly scanned at 2θ angles between 5 and 60°.

For the electrochemical impedance spectroscopy (EIS) analysis, the gels were placed in a custom-made sample holder and the impedance data were measured using a HIOKI 3532-50 LCR Hi-Tester with a frequency range of 50–5,000,000 Hz from 303 to 353 K. The corresponding ionic conductivity, σ was then calculated by the equation: σ = t/(RbA) where t is the thickness of the sample measured, A is the contact surface area and Rb is the bulk resistance of the sample. All the EIS measurements were repeated three times for each sample and the average value was calculated.

### 2.4. Characterization of Dye-Sensitized Solar Cell

The N719 dye coated TiO2 photo-anode was prepared by following an earlier reported procedure by Bandara et al. (Bandara et al., 2013). The platinum counter electrode used in this work was prepared by coating FTO glasses with Platisol T solution (from Solaronix SA) followed by sintering at 450 °C for 40 min. The DSSCs were fabricated by rod-coating the gels on the photoanode followed by sandwiching with the counter electrode. A 50 μm-thick spacer was used to maintain the electrolyte thickness. The performance of the QSDSSCs was studied using an Autolab instrument under solar simulator (Oriel LCS-100) with light intensity calibrated to 100 mW cm^−2^. The area of the tested DSSC was 0.196 cm^2^. Additionally, the DSSCs were tested with electrochemical impedance spectroscopy (EIS) using a potentiostat–galvanostat device (Metrohm Autolab PGSTAT128N, FRA32 M). The impedance of the cells was measured by applying a bias at the open-circuit voltage, V_OC_ of the cell and the readings were taken within a frequency range of 0.01–100,000 Hz with an a.c. amplitude of 10 mV.

## 3. Results

### 3.1. Rheological Properties

#### 3.1.1. Amplitude Sweep

**Figure 2:**
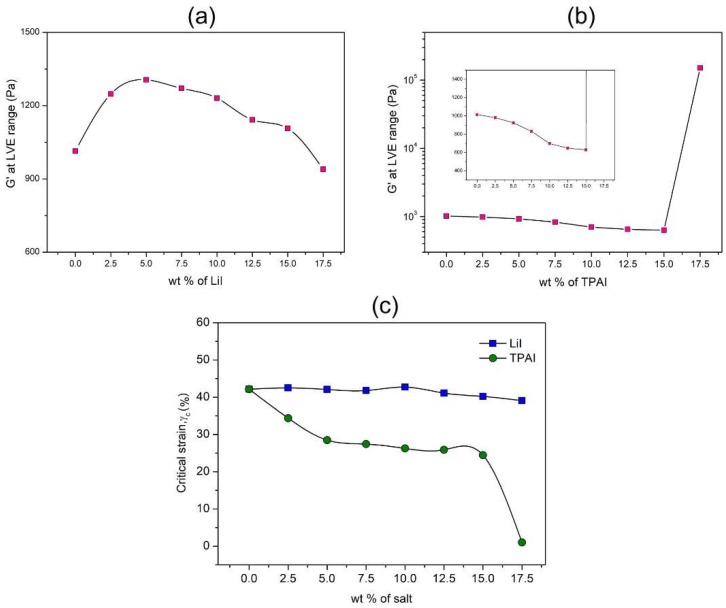
Storage modulus at the LVE range of (**a**) LiI and (**b**) TPAI based gel electrolytes and (**c**) critical strain values.

The amplitude sweep curves for PhSt-HEC-DMF gels with different amount of LiI and TPAI are attached in Figures S1 and S2. As depicted in Figure 2a, increasing amount of LiI content from 0 to 15 wt % resulted in an increment in the G’ value, which signifies improved stiffness. The enhanced rigidity of the gels with the addition of LiI could be the consequence of active interaction between the Li+ ions and the oxygen atoms present throughout the backbone of the polymers. The cation–polymer complexation enables a more compact packing of the polymer matrix framework, which gives rise to the solid character of the gels. This claim is further supported by the steady values of the critical strain of the gels, which shows that the gel strength was not affected significantly upon addition of LiI from 0 to 15 wt %. Beyond 15 wt %, the G’ value and critical strain shows a slight dip (Figure 2c). The high concentration of salt may have triggered formation of ion aggregates, thus lowering the possibility of the ion–polymer interaction.

Figure 2b shows the variation in G’ values for an increasing amount of TPAI. It is very interesting to note that the effect of TPAI addition on gel rigidity was the opposite of the effect induced by LiI. Similar observation has been reported by Dintcheva et al. who studied rheological behavior of PAN containing tetrahexyl ammonium iodide (THAI) and magnesium iodide (MgI_2_) salts [15]. They observed that PAN-EC-PC gel became more structured and rheologically stable by MgI_2_ loading. The increasing amount of TPAI from 0 to 15 wt % resulted in a significant drop of G’ values and the justification for this observation relies on the nature of the cation.

**Figure 3:**
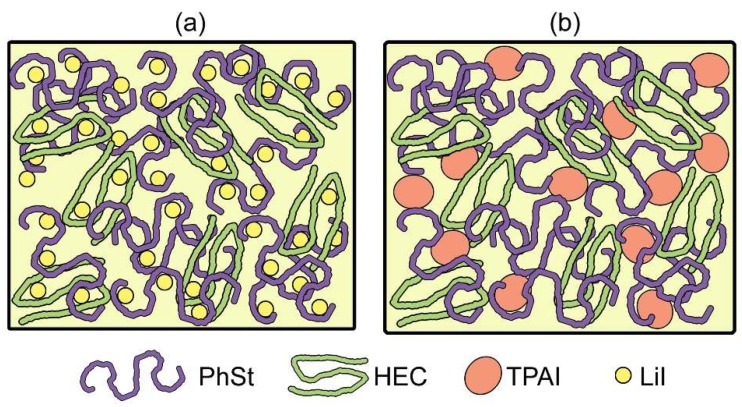
Graphical depiction of (**a**) Li+ and (**b**) TPA+ distribution in gel.

Lithium ions were relatively smaller in size and thus had the ability to extensively complex with the electronegative atoms of the polymer chains (Figure 3a). Unlike the tiny lithium ions, TPA^+^ was a bulky cation with limited mobility. The greater steric hindrance imposed by TPA^+^ disabled it from distributing itself extensively between the polymer chains (Figure 3b). In fact, the presence of these bulky cations amidst the polymer matrix is expected to prevent polymer chain entanglements. Thus, as the amount of TPA^+^ was increased, the rigidity of the gels continued to decline. However, upon loading of 17.5 wt % of TPAI, the G’ value at the linear viscoelastic range (LVE) range suddenly jumped to an elevated value (two orders of magnitude higher). This effect was due to recrystallization of the TPAI salt and the presence of the TPAI crystals in the gels gave rise to the extremely high solid character, which was translated as a high G’ value. As for the critical strain values that are depicted in Figure 2c, a drastic drop was observed with the initial introduction of TPAI (0–5.0 wt %). Between 5.0 and 15.0 wt %, a plateau was observed, signifying that despite the decreasing solid character, the strength of the gels remained unaffected at these concentrations. A sharp drop in critical strain value upon inclusion of 17.5 wt % of TPAI indicates that the homogeneity of the gel was highly disrupted. There was a high possibility of salt recrystallization at this concentration, which was also evident based on the unexpected high value of G’.

#### 3.1.2. Tack Test

**Figure 4:**
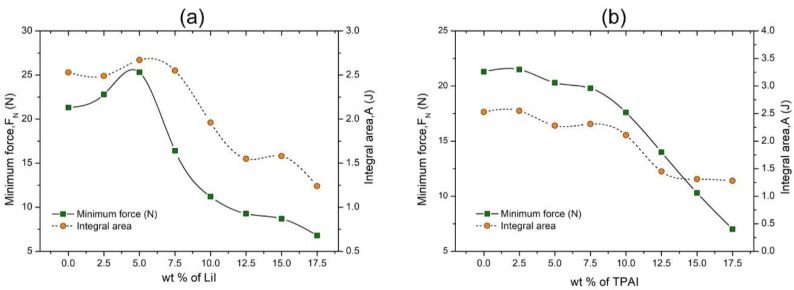
Tack test parameters of PhSt-HEC-DMF gels with (**a**) LiI and (**b**) TPAI.

Adhesiveness is the property of materials that are capable of forming an attachment with the substrate upon contact. This property is very vital for electrolytes in QSDSSC as it aids to establish effective contact between the electrodes and the electrolyte. The tack test curves obtained for different salt compositions are attached in Figure S3. As depicted in Figure 4a, the introduction of low quantity of LiI (from 0 to 5 wt %) impacted in improved adhesiveness signified by the higher force minimum values. The increment in the integral area under the curve verified that this was due to a higher cohesive and adhesive force. Beyond 5.0 wt % of LiI, both minimum force and integral area values began to drop. At high LiI concentration, the good adhesive property could not be imparted as effectively as at low concentration. On the other hand, addition of TPAI into the polymer gels resulted in a continuous downward trend in both the minimum force and integral area values (Figure 4b). The decline in adhesiveness was particularly obvious with addition of TPAI above 10 wt %. One of the contributing factors for this could be the downward trend in the storage modulus of these gels. In principle, adhesiveness is the product of balanced solid and liquid character of the gels. Thus, when the solid character continuously declines with addition of TPAI, this influences tan δ values, which in turn affects tackiness.

### 3.2. FTIR Analysis

Figure S4 depicts the trend in FTIR peaks for PhSt-HEC-DMF based gels with varying amounts of LiI and TPAI, respectively. Significant peak shifts could not be observed in all the electrolytes. Since the electrolytes consist of multiple components, the FTIR peaks observed are often a depiction of various overlapping peaks and thus, simple peak shift observations may not be useful here. In such a case, FTIR deconvolutions can be performed to reveal the presence of hidden peaks, which correspond to certain molecular interactions. In this work, the peaks of samples with 7.5 wt % of LiI and TPAI (L3 and T3) respectively, which are taken as representative of each electrolyte series, have been deconvoluted and analyzed. The chemical interactions within the electrolyte matrix were identified by analyzing the FTIR spectra in three main sections, namely, the ether stretching region (950–1150 cm^−1^), amide stretching region (1200–1550 cm^−1^) and carbonyl stretching region (1550–1750 cm^−1^), which were found to be particularly subjected to variations.

**Figure 5:**
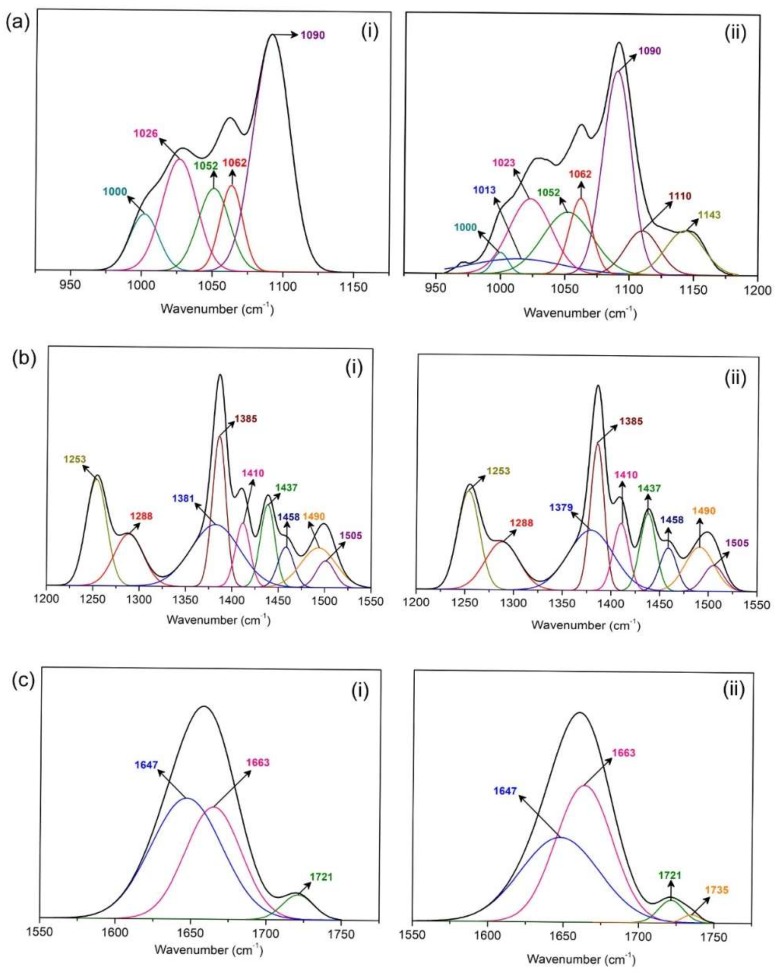
Deconvoluted FTIR spectra of PhSt-HEC-DMF with (i) LiI and (ii) TPAI gels in (**a**) ether, (**b**) amide and (**c**) carbonyl regions.

Within the range of 950–1150 cm^−1^ (Figure 5a), the contribution of the CH_3_ rocking mode of DMF at 1062 cm^−1^ and 1090 cm^−1^ are predominant in both L3 and T3 [16]. The peaks at 1052 cm^−1^ and 1026 cm^−1^, which attribute to the ether stretching in HEC and PhSt were also evident. In T3, additional peaks at 1000 cm^−1^, 1013 cm^−1^, 1110 cm^−1^, and 1143 cm^−1^, which correspond to interactions between ions and C-O groups were observed whereas in L3 only one additional peak at 1000 cm^−1^ was present. 

In the amide stretching region (Figure 5b), the peaks of both L3 and T3 resolve into nine individual peaks, most of which originate from DMF. The C-N stretching mode at 1505 cm^−1^, CH_3_ asymmetric deformation mode at 1437 cm^−1^, CH_3_ umbrella mode at 1410 cm^−1^, N-C-H bending mode at 1385 cm^−1^, and C-N asymmetric stretching at 1253 cm^−1^ can be clearly spotted [17,18]. The peak 1288 cm^−1^, on the other hand, corresponds to esteric C-O stretch. Besides this, three additional peaks were observed at 1379 cm^−1^, 1458 cm^−1^, and 1490 cm^−1^, which are presumed to be downshifted peaks of N-C-H bending mode and C-N stretching respectively. The coordination of cations to the lone pair of the nitrogen accounts for the manifestation of these peaks at a lower wavenumber [19].

As for the range of the wavenumber between 1550 and 1750 cm^−1^ (Figure 5c), the region was overshadowed by two main peaks at 1663 cm^−1^ and 1721 cm^−1^ attributed to C=O stretching in DMF and PhSt. Additional peak at 1647 cm^−1^ presumably due to the coordination of cations and the carbonyl group was also present in both L3 and T3. Interestingly, a tiny peak at 1735 cm^−1^ was existent in T3. As this peak appeared at a higher wavenumber than the original carbonyl peak, which was at 1721 cm^−1^, the origin of this peak was speculated to be an interaction between the carbonyl in PhSt and anions [19].

### 3.3. Crystallinity

**Figure 6:**
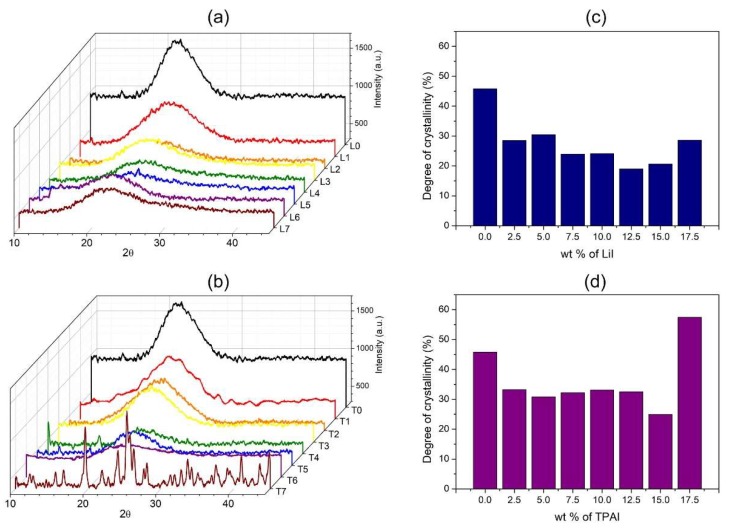
XRD diffractograms of (**a**) LiI and (**c**) TPAI and degree of crystallinity of (**b**) LiI and (**d**) TPAI based electrolytes.

From the diffractograms of the electrolytes as shown in Figure 6, incorporation of small amount of LiI and TPAI resulted in drastic reduction of crystallinity of the polymer gels. Introduction of cations and anions into the polymer matrix disrupts the structural orderliness of the polymer chain arrangement. As shown in Figure 6b, the increment of LiI content induced a gradual decline in degree of crystallinity up to 12.5 wt % of salt. This trend concurs with the observations in the rheological analysis, which connoted active complexation between Li^+^ and the oxygen in the polymer backbone. The interaction between ion and polymer occupies the electronegative oxygen atoms throughout the polymer backbone. This in turn deters polymer–polymer H bonding, which is often the main source of the crystallization in polysaccharides.

The initial incorporation of TPAI salt into the polymer gel resulted in a drastic reduction of crystallinity (Figure 6d). However, from 2.5 to 12.5 wt % of TPAI, the degree of crystallinity remained within similar values. As the bulky TPAI cations were more likely to be stranded within the polymer matrix, it would not be as effective as the Li^+^ ions in forming ion-polymer complexation. Hence, the addition of TPAI beyond a certain amount results in positive effects towards the crystallinity of the polymer gels. Addition of the salt beyond 12.5 wt % caused increased crystallinity in both salt systems. For LiI series, the excessive amount of ions was expected to form ion aggregates, thus reducing the possibility of the ion–polymer interaction. In the case of TPAI series, gels with salt concentration of 17.5 wt % manifested intense sharp peaks in their XRD diffractograms, which coincided with the peaks of pristine TPAI. This peaks established the recrystallizations of TPAI in those gels, which was also reflected by the sudden increase in the degree of crystallization at 17.5 wt % TPAI. 

### 3.4. Electrochemical Properties

**Figure 7:**
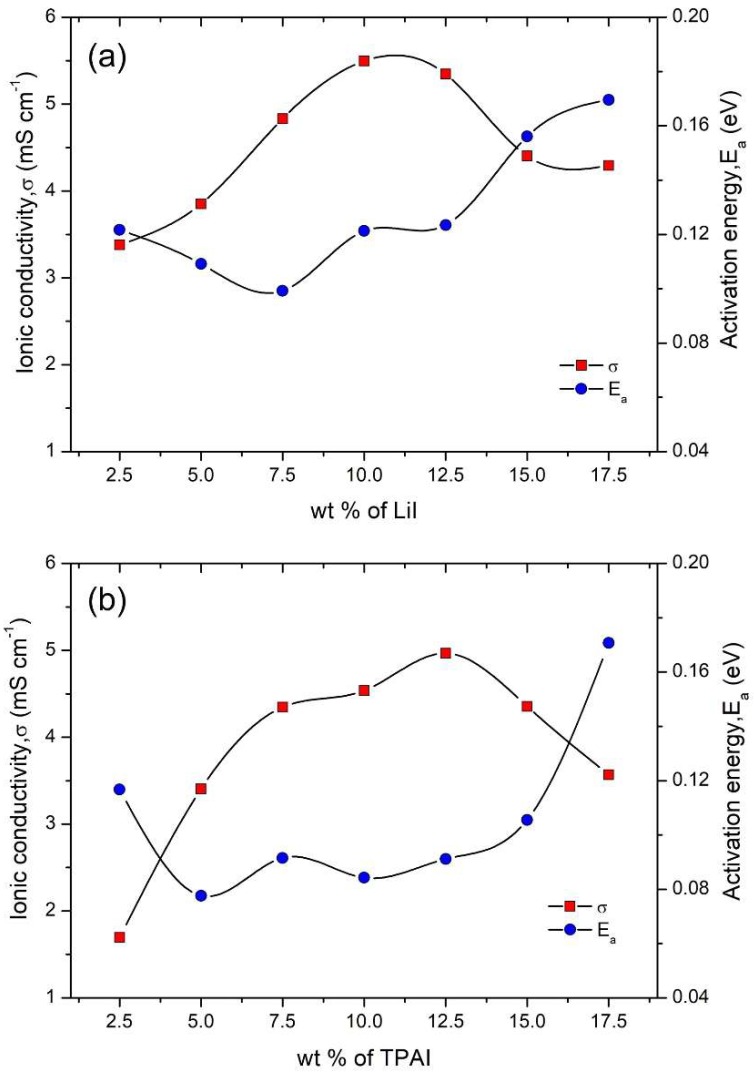
Ionic conductivity (at 30 °C) and activation energy of PhSt-HEC-DMF with (**a**) LiI and (**b**) TPAI gels.

Figure 7 portrays the variation of ionic conductivities, σ (at 30 °C) as a function of LiI and TPAI content, respectively. In the case of both the salts, the ionic conductivities increase with a higher amount of salt, eventually reaching an optimum value and then drops. This pattern was typically found in most polymer electrolytes. In the LiI series as shown in Figure 7a, the highest σ of 5.50 × 10^−3^ S cm^−1^ was attained with the inclusion of 10 wt % of LiI whereas, in the TPAI series, a maximum conductivity of 4.97 × 10^−3^ S cm^−1^ was achieved by 12.5 wt % of TPAI (Figure 7b). The gradual increase in σ up to an optimum salt composition was attributed to the increase in the number of mobile charge carriers in the electrolytes. When the amount of salt exceeded this optimum salt content, the excessive presence of ions tended to create steric crowding, which might also result in reassociation or polyionization [20]. 

The effect of salt composition on σ is found to be less dependent on factors affecting the polymeric structure such as crystallinity and stiffness of the gels. This can be viewed under two perspectives. Firstly, the difference in the magnitude of the storage moduli and the variation in crystallinity of the gels with the addition of salt is not as prominent as observed in different PhSt-HEC compositions. Secondly, the ion-conduction in a quasi-solid electrolyte, as it is the case in the current work, occurs via the local solvent channels built around the polymer gel network. Thus, the ion transfer is primarily influenced by the hopping of these ions through the solvent channels rather than segmental mobility of polymer chains. Therefore, the domination of type and amount of ions in electrolytic solution is more superior in affecting σ as compared to the arrangement of the polymer network.

The dominant ion conduction mechanism of polymer electrolytes can be identified by monitoring the temperature-dependent conductivity behavior of the samples. If the temperature dependence of ionic conductivity obeys the Vogel–Tamman–Fulcher (VTF) equation, then ion migration is strongly governed by segmental motion of the polymer [21]. On the other hand, if the temperature-dependent conductivity plot complies with Arrhenian behavior, then ion transport is attributed to ion hopping via the solvent channels. PhSt-HEC based gels with varying amounts of TPAI and LiI demonstrated conformation to Arrhenius behavior. As evident in Figure 7, in both LiI and TPAI series, the E_a_ values oscillates within a small range of values (0.08–0.12 eV) for low and moderate salt concentrations. A prominent rise in E_a_ value can only be observed when the LiI and TPAI salt is in high amount (beyond 15 wt %). Recent reports have highlighted that at low and moderate salt concentrations, the E_a_ value is largely independent of the salt concentration [22]. This is again attributed to the ion conduction mechanism through solvent channels, thus causing the ion transport to be dominated by a single activation process, which substantially depends on the solvent family.

### 3.5. Photovoltaic Performances

**Figure 8:**
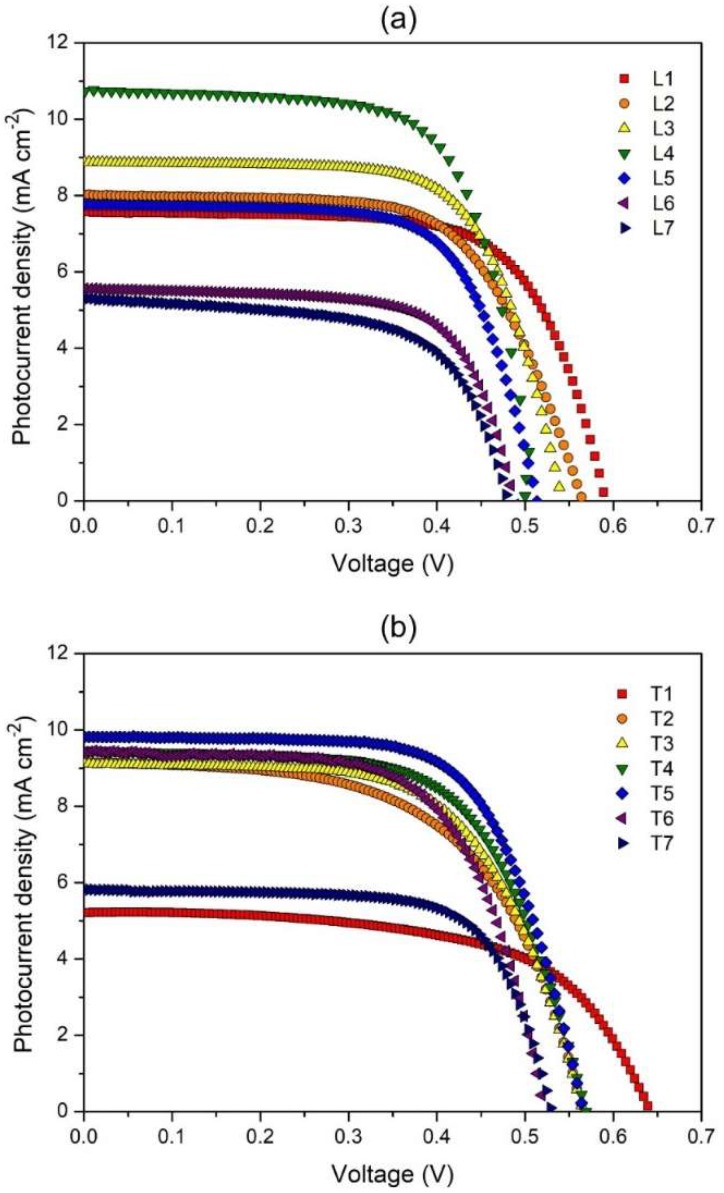
JV curves of PhSt-HEC-DMF with (**a**) LiI and (**b**) TPAI gels.

**Figure 9:**
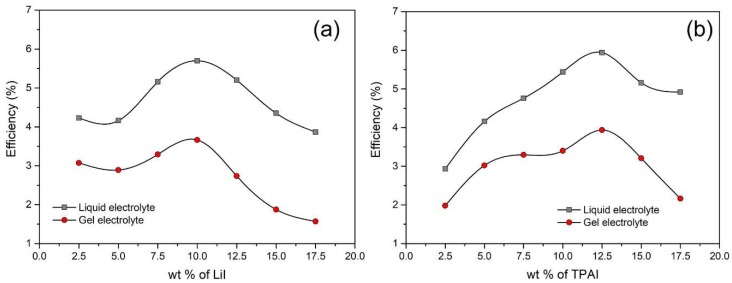
Comparison in the efficiency of the (**a**) liquid electrolyte and (**b**) quasi-solid electrolyte.

Figure 8 depicts the J–V curve obtained from the DSSC fabrication of the gel electrolytes. Figure 9 summarizes the trend in photoconversion efficiencies of both salt systems and for comparison, solar cells comprising of only the liquid electrolyte (LE) counterpart of each designation were also fabricated. The general trend in efficiency was found to be similar for both liquid and gel electrolyte systems. As expected, the LE system exhibited higher efficiency relative to the GPE and this observation is presumably due to better mobility of ions in the liquid state compared to the gel state.

In the LiI series, a maximum value of photoconversion efficiency of 3.67% was obtained with the addition of 10.0 wt % LiI (Table 2). The trend in J_SC_ values showed increment up to 10.0 wt % LiI contributed by the increasing amount of iodide ions. However, beyond 10.0 wt % of LiI, the short circuit current values began to drop and this was proposed to be the effect of higher concentration of Li^+^ ions within the gel, which might restrict the mobility of iodide ions. The open circuit voltage, on the other hand, exhibited a continuous downward trend as the amount of LiI increased. This is because, small cations such as Li^+^ have the tendency to be adsorbed onto the TiO_2_ layer in the photoanode and this alters the flat band potentials [23]. The fill factor values for all the cells in this series are very similar, lying between 65% and 69%. Therefore, efficiency is mainly governed by the J_SC_ and the V_OC_ values. Between 2.5 and 10.0 wt % of LiI, there is a drastic drop in V_OC_ accompanied by a sharp increase in Jsc. Both these phenomena are related to each other, as when the flat band potential of the semiconductor is lowered, the driving force for the electron injection from dye to TiO_2_ increases [24]. The higher injection efficiency, η_inj_ will sequentially improve J_SC_ values. Therefore, in these range of LiI compositions, the tug of war between J_SC_ and V_OC_ affected efficiency value adversely. Beyond 10 wt % LiI, the decline in both J_SC_ and V_OC_ values cumulatively lead to a sharp dip in the solar cell efficiencies. 

For the TPAI series, the highest efficiency of 3.94% was attained upon inclusion of 12.5 wt % of TPAI (Table 2) and this is one of the best DSSC performances in comparison to other starch based electrolytes in recent literature. J_SC_ values increased with higher amount of TPAI up to 12.5 wt %, more than which the values declined presumably due to ion aggregation. There was an initial drop in the V_OC_ value between 2.5 and 5.0 wt % TPAI but from 5 wt % onwards the values remained steady. Only when TPAI salt concentration was higher than 12.5 wt %, there was a perceptible decline of V_OC_ due to the cation adsorption. Fill factor values were found to be situated between 60% and 69%. The overall pattern in efficiency for the TPAI series was heavily dependent on the J_SC_ values. Interestingly, the highest J_SC_ in the TPAI series was in fact lower than that in the LiI series. Nevertheless, the ability of TPAI based electrolytes to sustain stable V_OC_ values yielded solar cell with better photoconversion efficiency (about 7% higher than LiI). Besides the small difference in performance, TPAI salt based gels are also generally preferred over LiI due to production sustainability. This is because lithium salt has adverse effects in terms of long term availability and cost. 

It is noteworthy to mention that the variation in J_SC_ of the TPAI series matched the trend in the ionic conductivities of these gels. However, in the LiI series, the J_SC_ values did not concur with the ionic conductivity values. These observations reflect the contribution of ionic species in the conductivity of both salt systems. When TPAI is employed as the charge carrier, the bulky TPAI is presumed to be entangled in the polymer matrix, allowing iodide ion mobility to mainly contribute to the overall ionic conductivity of the gel. Whereas, in the case of LiI, conductivity is the outcome of the motion of both Li^+^ and I^−^ ions. Therefore, high conductivity does not necessarily translate into improved iodide ion transport. Typically, the impact of cation on the Jsc values in a DSSC can either be related to the difference in overall conductivity of the triiodide species or to the changes in the charge injection rate from the dye to the semiconductor, which can be altered via cation adsorption. In this work, it is evident that for LiI series, the Jsc values were impacted on the basis of ion adsorption, which reduced the Voc values, providing opportunity for greater electron injection rates between the dye and semiconductor. On the other hand, in the TPAI series, Jsc values were more dependent on the overall ionic conductivity of the electrolytes, as high conductivity hints enhanced iodide ion mobility.

The maximum photoconversion reported in this work is relatively higher than the value obtained by contemporary studies on starch based DSSC (Table 3). This shows that the blending of starch and cellulose as well as the employment of appropriate quantity of a large cation iodide salt are effective measures to improve the performance of the cell. To further comprehend how the biopolymer electrolyte affect interfacial processes, the cells were subjected to EIS measurements.

### 3.6. Impedance Study of DSSC

**Figure 10:**
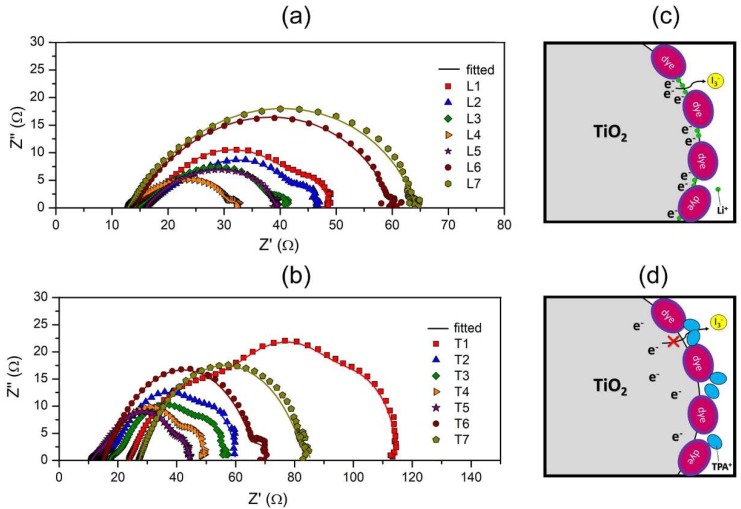
Nyquist plots of quasi-solid DSSC (QSDSSC) fabricated using (**a**) LiI and (**b**) TPAI gels and illustration of cation interaction with photoanode surface for (**c**) LiI and (**d**) TPAI gels.

In both the series, the variation of salt content affected the impedance at each interface. The Nyquist plot of the DSSC fabricated generally exhibited three semicircles as depicted in Figure 10. The first semicircle appearing at a higher frequency is attributed to the triiodide reduction at the counter electrode (R_PT_). Thus a lower value of R_PT_ signifies an efficient electrocatalytic activity at the platinum electrode. As tabulated in Table 4, in the LiI series, the R_PT_ values between L1 and L4 were in the lower range (<10 Ω). This is seen to be a repercussion of the high conductivity of these electrolytes, which ensure rapid ion transport. Therefore, beyond L5, where a decline in ionic conductivity was also evident, the R_PT_ values escalated drastically. Similar observations were projected in the TPAI series, in which trends of σ and R_PT_ were the inverse to each other. 

The second semicircle in the Nyquist plots addresses the charge recombination reaction between the photoanode and TiO_2_ as represented in the equation below. Suppressing this reaction is important to maximize the overall efficiency and thus larger R_CT_ will benefit the performance of the cell. In both LiI and TPAI series, increasing the salt content resulted in the reduction of R_CT_ values. This trend can be associated with the diminishing adhesive nature of the gels as highlighted in the tack test analysis. Particularly, in the LiI series, an appreciable decline in the R_CT_ was only observed beyond 10 wt %, the same region at which the minimum force of tack test drastically reduced. These findings emphasize that if the electrolyte employed possess good tack behavior to optimize the contact between electrodes, then suppression of charge recombination between photoanode and I_3−_ occurs more effectively.

Another interesting pattern noticed between the two series was that, predominantly, the R_CT_ values of TPAI series was larger than LiI. The steric hindrance imposed by TPAI limits their penetration into the sensitized photoanode (Figure 10c,d). This in turn minimizes the screening effect of negative charge at the TiO_2_/electrolyte interface and successively diminishes the local concentration of counter anions, I_3−_. Therefore the possibility of electron recombination from TiO_2_ and I_3−_ in the electrolyte is lower [28].

The third semicircle in the Nyquist plot appearing at the lowest frequency domain correlates to the diffusion of the redox couple in the electrolyte. From Figure 10 it is apparent that for samples L5 to L7 and T7 the third semicircle is not present. It is suspected that for these samples, the impedance attributed to the Nernst diffusion process features at frequencies lower than the minimum limit of the instrument used (0.01 Hz). Nevertheless, by analyzing the electrolytes with the third semicircle, it was evident that R_D_ and $D_{{I_{3}}^{-}}$ values decreased with higher salt concentration and concurred with the conductivity values of the gels.

Overall, the sheet resistances of all the samples were found to be between 11 and 17 Ω except for T1 and T7, which showed unexceptionally high R_S_ values. The anomaly in the R_S_ values of T1 and T7 is attributed to poor ionic σ and recrystallization of salt respectively.

## 4. Conclusions

In this study, the PhSt-HEC blend based quasi-solid electrolyte was fabricated by utilizing LiI and TPAI respectively, representing a small and bulky cation of iodide salt. The rheological and electrochemical properties of the gels were optimized by varying the composition of each salt. The size of the cation was found to significantly affect rheological properties of the resulting gels. Addition of LiI enhanced the rigidity and tackiness of the gels, whereas TPAI inclusion resulted in an opposing effect. Gel composition with 12.5 wt % TPAI generated the highest photoconversion efficiency of 3.94%. The good balance of high J_SC_ and V_OC_ values enabled the TPAI based electrolyte to manifest better photovoltaic performance than LiI. EIS revealed higher charge transfer resistance observed in gels with TPAI compared to LiI, indicating the possibility of bulky cations’ ability to suppress recombination reaction at the electrode–electrolyte interface.

## Figures and Tables

**Table 1:** Designation of phthaloyl starch and the HEC based electrolyte.

Designation		Mass of Salt (g)	Wt % of Salt (%)	Mass of I_2_ (g)
L Series	T Series			
L0	T0	0.00	0.0	0.000
L1	T1	0.04	2.5	0.008
L2	T2	0.08	5.0	0.016
L3	T3	0.13	7.5	0.025
L4	T4	0.18	10.0	0.034
L5	T5	0.23	12.5	0.043
L6	T6	0.28	15.0	0.054
L7	T7	0.34	17.5	0.064

**Table 2:** JV parameters of phthaloyl starch and the HEC based electrolyte.

Sample	wt % of Salt	σ (×10^−3^ S·cm^−1^)	η (%)	J_SC_ (mA cm^−2^)	V_OC_ (V)	FF
LiI series						
L1	2.5	3.38	3.07	7.69	0.59	0.68
L2	5.0	3.85	2.89	8.13	0.56	0.67
L3	7.5	4.83	3.29	8.85	0.54	0.69
L4	10.0	5.50	3.67	10.68	0.51	0.65
L5	12.5	5.35	2.74	7.77	0.51	0.68
L6	15.0	4.40	1.87	5.78	0.48	0.67
L7	17.5	4.29	1.57	5.03	0.48	0.65
TPAI series						
T1	2.5	1.69	1.98	5.15	0.61	0.63
T2	5.0	3.41	3.02	9.02	0.57	0.60
T3	7.5	4.35	3.29	9.16	0.57	0.60
T4	10.0	4.54	3.40	9.46	0.56	0.64
T5	12.5	4.97	3.94	10.11	0.56	0.69
T6	15.0	4.35	3.21	8.92	0.52	0.68
T7	17.5	3.57	2.16	5.82	0.52	0.71

**Table 3:** Recent studies on starch based dye-sensitized solar cell (DSSC).

Electrolyte Composition (Polymer/Salt/Additive)	Dye	η (%)	J_SC_ (mA cm^−2^)	V_OC_ (V)	FF	Ref.
**Rice starch/LiI/MPII//TiO**_**2**_	N3	0.17	0.49	0.45	0.75	[7]
**Rice starch/LiI/Distilled water**	N719	0.35	0.83	0.92	0.46	[25]
**GMIC grafted starch/KI/DMSO**	N719	0.63	0.49	0.55	0.61	[8]
**Rice starch/NaI**	N719	0.78	2.40	0.49	0.67	[6]
**Crosslinked starch/LiI/Gly/DMF**	N719	1.40	2.17	0.67	0.82	[9]
**Rice starch/NaI/MPII**	N719	2.09	4.78	0.57	0.77	[26]
**PhSt/HEC/LiI/DMF**	N719	3.02	9.02	0.57	0.60	[11]
**Potato starch nanocrystal/DMSO/NaI**	N719	3.33	8.08	0.72	0.57	[27]
**PhSt/HEC/LiI/DMF**	N719	3.94	10.11	0.56	0.69	This work

Table 3 abbreviation list: LiI: lithium iodide, MPII: 1-methyl-3-propylimidazolium iodide, TiO_2_: titanium dioxide, GMIC: 1-glycidyl-3-methylimidazolium chloride, KI: potassium iodide, DMSO: N,N-dimethylsulfoxide, NaI: sodium iodide, Gly: glycerol and DMF:N,N-dimethylformamide.

**Table 4:** Equivalent circuit parameters of phthaloyl starch and the HEC based electrolyte.

Designation	R_S_ (Ω)	R_PT_ (Ω)	R_CT_ (Ω)	R_D_ (Ω)	$D_{{I_{3}}^{-}}$ (×10^−7^ cm^2^ s^−1^)	Σ (×10^−3^ S cm^−1^)
LiI series						
L1	13.92	8.17	18.58	8.29	6.71	3.38
L2	15.91	8.12	17.62	5.48	6.46	3.85
L3	14.78	5.88	17.58	3.26	7.73	4.83
L4	12.68	6.36	11.97	1.89	8.76	5.50
L5	16.11	12.07	11.23	-	-	5.35
L6	13.21	32.90	12.95	-	-	4.40
L7	12.81	39.80	10.42	-	-	4.29
TPAI series						
T1	23.79	24.15	41.82	25.12	6.58	1.69
T2	13.43	9.16	22.59	15.72	7.23	3.41
T3	17.06	6.00	22.56	11.99	7.33	4.35
T4	12.37	5.62	21.58	9.00	8.38	4.54
T5	11.10	5.11	23.05	4.49	11.30	4.97
T6	15.03	23.50	26.47	6.09	4.15	4.35
T7	27.00	39.05	20.66	-	-	3.57

## Supplementary Materials

The following are available online at https://www.mdpi.com/2073-4360/12/3/516/s1, Figure S1: Amplitude sweep curves of PhSt-HEC-DMF gel electrolytes with LiI; Figure S2: Amplitude sweep curves of PhSt-HEC-DMF gel electrolytes with TPAI; Figure S3: Tack test curves of PhSt-HEC-DMF gel electrolytes with (a) LiI and (b) TPAI; Figure S4: FTIR spectra of PhSt-HEC-DMF gel electrolytes with (a) LiI and (b) TPAI.
