# Supplementary material for: Organosoluble Starch-Cellulose Binary Polymer Blend as a Quasi-Solid Electrolyte in a Dye-Sensitized Solar Cell
Source: Polymers (Basel). 2020 Feb 27;12(3):516. doi: 10.3390/polym12030516 (PMC7182809; doi:10.3390/polym12030516)
Supplement: Supplementary file 1 [file polymers-12-00516-s001.pdf]

Organosoluble Starch-Cellulose Binary Polymer Blend as a Quasi-Solid Electrolyte in a Dye-Sensitized Solar Cell

Vidhya Selvanathan [1,*], Rosiyah Yahya [2], Mohd Hafidz Ruslan [1], Kamaruzzaman Sopian [1], Nowshad Amin [3], Majid Nour [4], Hatem Sindi [4], Muhyaddin Rawa [5] and Md. Akhtaruzzaman [1,6,*]

[1] Solar Energy Research Institute (SERI), Universiti Kebangsaan Malaysia (UKM), Bangi 43600, Selangor Darul Ehsan, Malaysia; hafidzruslan@gmail.com (M.H.R.); ksopian@ukm.edu.my (K.S.)

[2] Department of Chemistry, Faculty of Science, University of Malaya, Kuala Lumpur 50603, Malaysia; rosiyah@um.edu.my

[3] Institute of Sustainable Energy, Universiti Tenaga Nasional (@The National Energy University), Jalan IKRAM-UNITEN, 43000 Kajang, Selangor, Malaysia; nowshad@uniten.edu.my

[4] Department of Electrical and Computer Engineering, King Abdulaziz University, Jeddah 21589, Saudi Arabia; mnour@kau.edu.sa (M.N.); hfsindi@kau.edu.sa (H.S.)

[5] Centre of Research Excellence in Renewable Energy and Power Systems, King Abdulaziz University, Jeddah 21589, Saudi Arabia; mrawa@kau.edu.sa

[6] Department of Electrical, Electronic and Systems Engineering, Faculty of Engineering and Built Environment, The National University of Malaysia, 43600 Bangi, Selangor, Malaysia

* Correspondence: Correspondence: vidhya.uchem@gmail.com (V.S.); akhtar@ukm.edu.my (M.A.)

Received: 29 January 2020; Accepted: 24 February 2020; Published: date

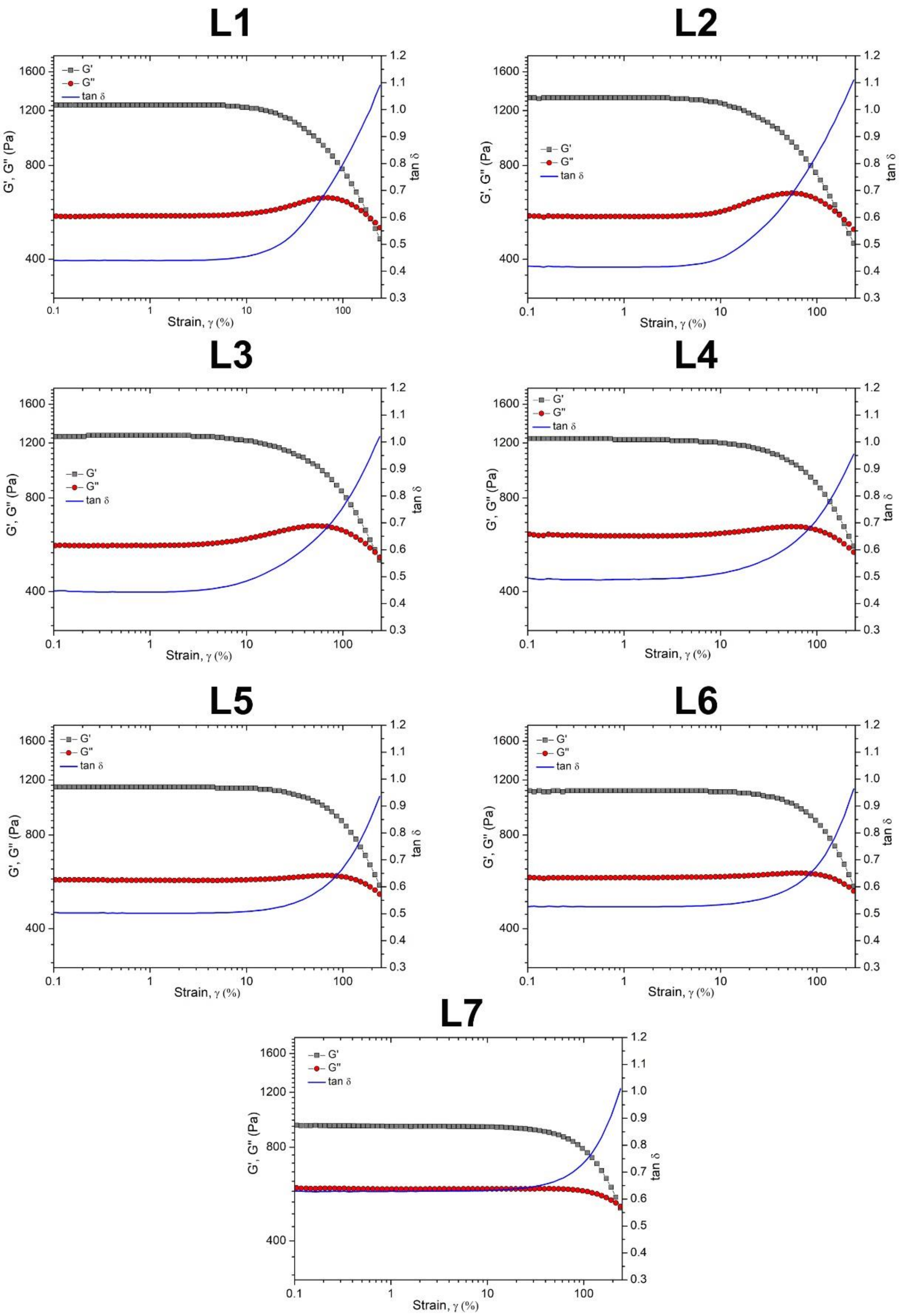

Figure S1: Amplitude sweep curves of PhSt-HEC-DMF gel electrolytes with LiI

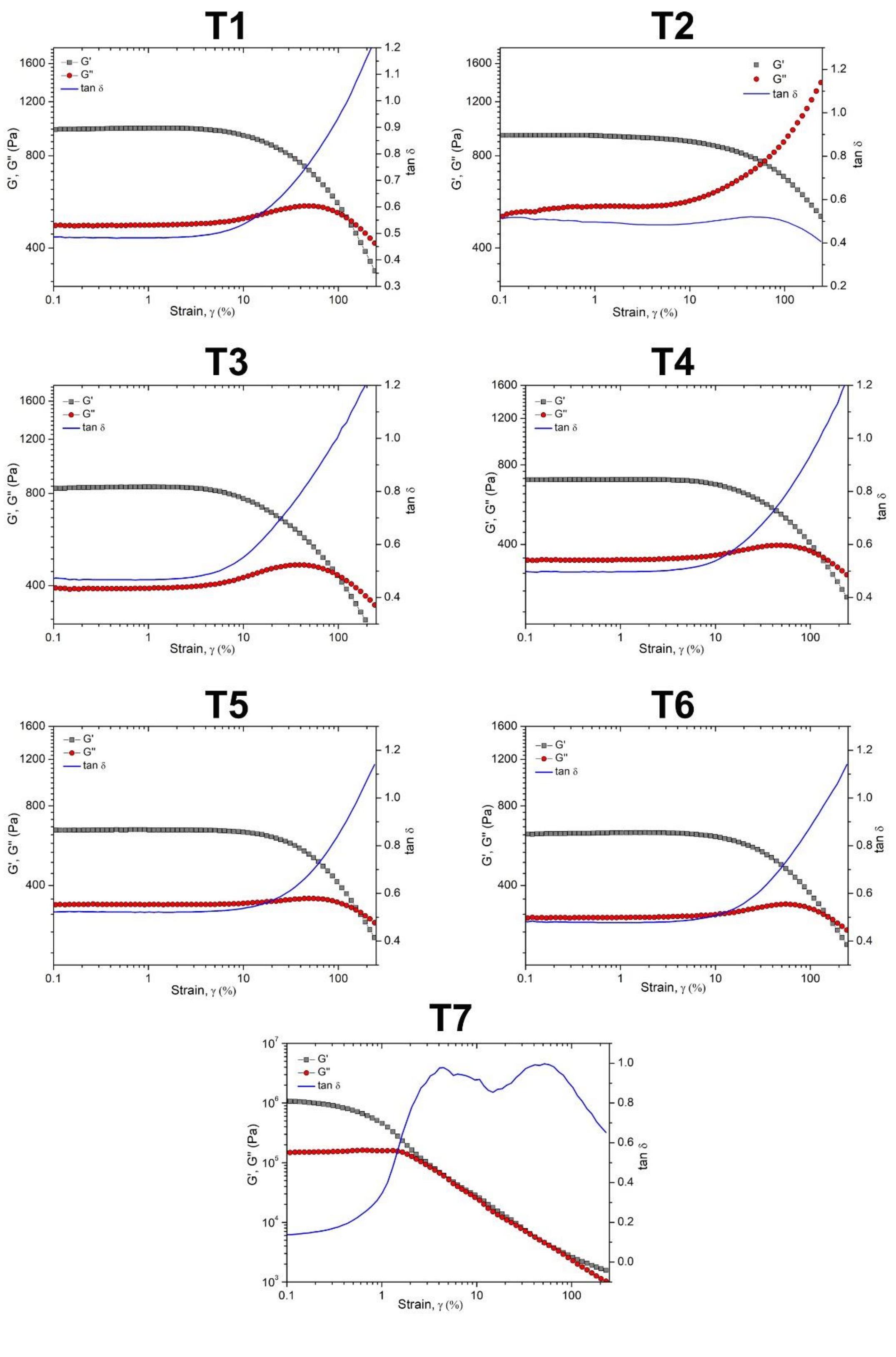

Figure S2: Amplitude sweep curves of PhSt-HEC-DMF gel electrolytes with TPAI

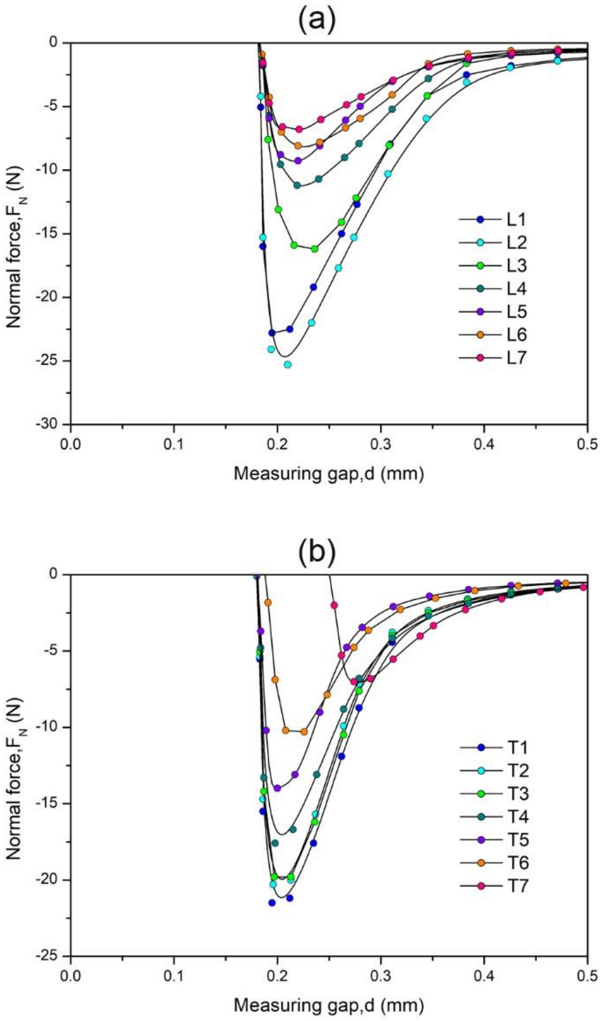

Figure S3: Tack test curves of PhSt-HEC-DMF gel electrolytes with (a) LiI and (b) TPAI

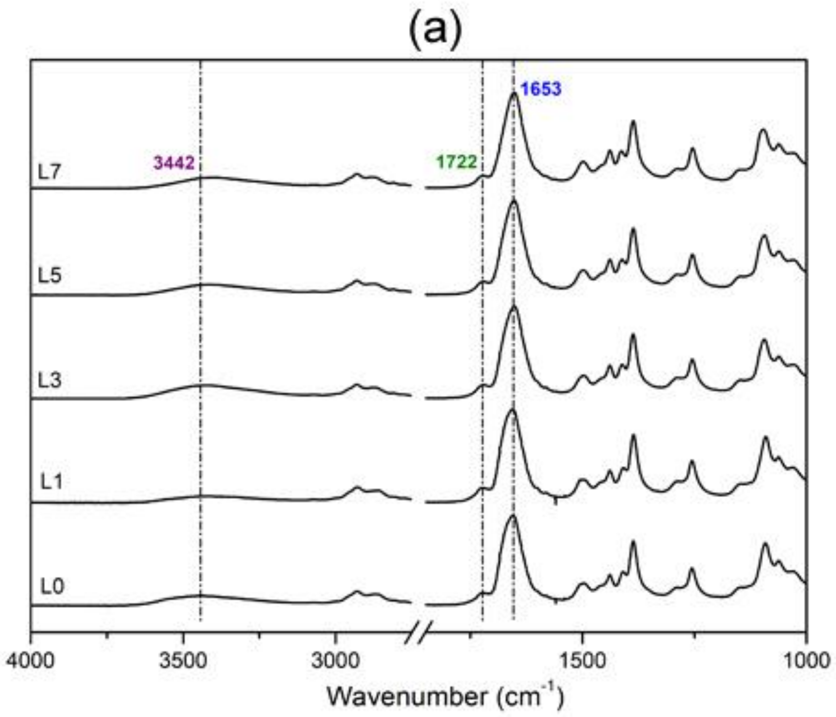

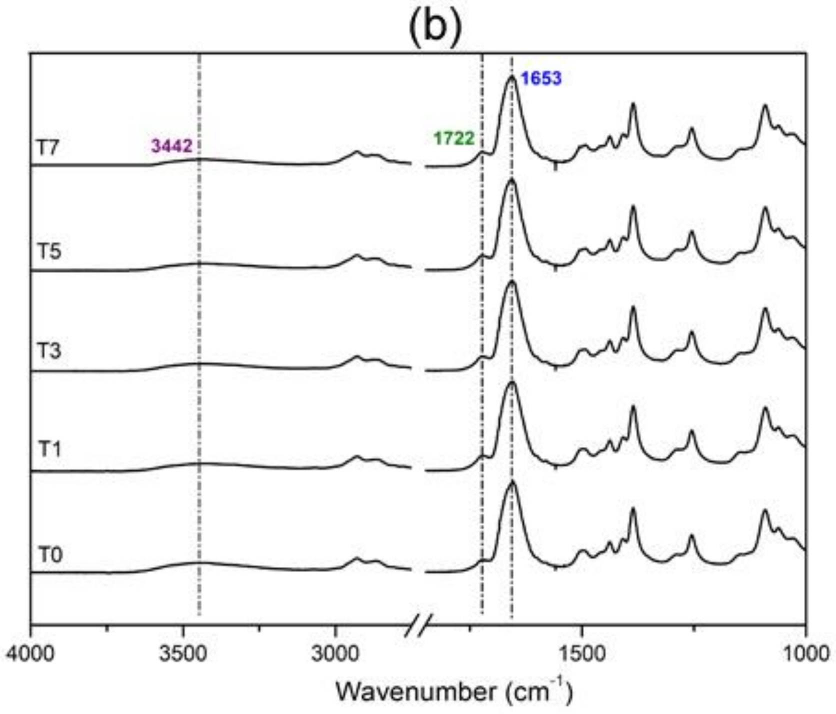

Figure S4: FTIR spectra of PhSt-HEC-DMF gel electrolytes with (a) LiI and (b) TPAI
